# Strategies to Preserve Cognition in Patients With Brain Metastases: A Review

**DOI:** 10.3389/fonc.2018.00415

**Published:** 2018-10-09

**Authors:** Tyler P. Robin, Chad G. Rusthoven

**Affiliations:** Department of Radiation Oncology, University of Colorado School of Medicine, Aurora, CO, United States

**Keywords:** brain metastases (BM), radiosurgery, cognition, neurocognition, whole brain radiation therapy (WBRT), hippocampus, memantine, tyrosine kinase inhibitor

## Abstract

Brain metastases are common to the natural history of many advanced malignancies. Historically, whole brain radiation therapy (WBRT) has played a key role in the management of brain metastases, especially for patients with multiple lesions. However, prospective trials have demonstrated consistent neurocognitive toxicities after WBRT, and various pharmacologic and anatomic strategies designed to mitigate these toxicities have been studied in recent years. Memantine, an NMDA receptor antagonist, taken during and after WBRT improved cognitive preservation in a randomized trial over placebo. Deliberate reductions in radiation dose to the hippocampus, via hippocampal-avoidance (HA)-WBRT, resulted in improved cognition over historic controls in a phase II trial, and follow-up randomized trials are now ongoing to evaluate cognitive outcomes with HA vs. conventional brain radiation techniques. Nevertheless, some of the most promising strategies currently available to reduce the cognitive effects of brain radiation may be found in efforts to avoid or delay WBRT administration altogether. Stereotactic radiosurgery (SRS), involving focused, high-dose radiation to central nervous system (CNS) lesions with maximal sparing of normal brain parenchyma, has become the standard for limited brain metastases (classically 1–3 or 4 lesions) in the wake of multiple randomized trials demonstrating equivalent survival and improved cognition with SRS alone compared to SRS plus WBRT. Today, there is growing evidence to support SRS alone for multiple (≥4) brain metastases, with comparable survival to SRS alone in patients with fewer lesions. In patients with small-cell lung cancer, the routine use of prophylactic cranial irradiation (PCI) for extensive-stage disease has been also been challenged following the results of a randomized trial supporting an alternative strategy of MRI brain surveillance and early salvage radiation for the development of brain metastases. Moreover, new systemic agents are demonstrating increasing CNS penetration and activity, with the potential to offer greater control of widespread and microscopic brain disease that was previously only achievable with WBRT. In this review, we endeavor to put these clinical data on cognition and brain metastases into historical context and to survey the evolving landscape of strategies to improve future outcomes.

## Introduction

Paradigms for the management of brain metastases are evolving, with increasing treatment options and a greater focus on cognitive preservation. In an effort to mitigate the neurocognitive effects of whole brain radiation (WBRT) and prophylactic cranial irradiation (PCI), both anatomic and pharmacologic strategies have been studied in recent years, including hippocampal-avoidance radiation and the concomitant use of the drug memantine for neuroprotection (1, 2). In addition, one of the most promising neurocognitive preservation strategies has been the more limited use of WBRT and PCI altogether (3). There is growing evidence to support the use of stereotactic radiosurgery (SRS) for patients with multiple brain metastases, and guideline statements have been adapted to reduce strict reliance on lesion number for selection of SRS candidates (4–6). The contemporary role of PCI for small-cell lung cancer in the era of MRI staging and surveillance has also been challenged by a recent randomized trial (7). Concurrently, a number of emerging systemic therapies have shown increasing CNS penetration and activity, blurring the historic lines of distinction between anticipated CNS and extra-CNS disease response rates to systemic therapy (8–20). Herein, we review the emerging clinical data on neuroprotective strategies and attempt to place these data into the historical context of brain metastases management.

## WBRT: CNS disease control, cognition, and survival

WBRT has been the historic standard for the management of brain metastases and, prior to the more widespread availability of SRS, WBRT often represented the only means for treating unresected brain metastases in cases ranging from diffuse to solitary CNS lesions. As access to SRS technology increased a number of trials began comparing strategies of SRS alone to SRS plus WBRT for limited (1–3 or 4) brain metastases (21–25). The results of these trials, detailed below, would ultimately make SRS alone the contemporary standard of care for limited brain metastases; however, the role of SRS alone in multiple (often defined as ≥4) lesions remains somewhat controversial due to the exclusion of these patients from the landmark randomized trials (5). In addition, WBRT delivered in the form of PCI for patients without evidence of brain metastases remains the standard of care for patients with limited-stage small-cell lung cancer (LS-SCLC) following a response to first-line therapy, and is an option for patients with extensive-stage (ES) disease (26). Thus, the neurocognitive impact of WBRT and PCI remain highly relevant to contemporary clinical practice.

Multiple randomized trials of SRS alone vs. SRS plus WBRT for patients with limited metastases have demonstrated that, overall, the addition of WBRT is associated with (1) objective declines in neurocognitive function, (2) improved CNS disease control rates, but (3) no benefit in terms of OS (21–25). The first major trial published was a multicenter Japanese study reported by Aoyama et al. in 2006. That study randomized 132 patients with 1–4 brain metastases to WBRT and SRS or SRS alone and found an improvement in CNS control rates with no differences in OS with the addition of WBRT (21). Similarly, an EORTC trial enrolled patients with 1–3 brain metastases treated initially with SRS or surgical resection (local therapy was at the physician's discretion) and randomized them to WBRT or observation. This trial also observed a reduction in CNS progression events with WBRT, but no differences in OS (23). While these trials clearly demonstrated that WBRT did not significantly affect OS outcomes, the collection of rigorous cognitive data was limited. In a single-institution phase III trial at MD Anderson, Chang et al. randomized patients with 1–3 brain metastases to SRS alone vs. SRS plus WBRT with a primary endpoint of neurocognitive function. This study was stopped early by the data safety monitoring committee due to increased cognitive decline on the Hopkins Verbal Learning Test-Revised (HVLT-R) total recall at 4 months with WBRT. For SRS plus WBRT, the mean probability of decline in total recall, delayed recall, and delayed recognition, was 52, 22, and 11%, respectively, compared with 24, 6, and 0% for patients treated with SRS alone (22). In an NCCTG study, Brown et al. reported the results of a randomized trial comparing SRS alone to SRS plus WBRT for 1–3 brain metastases with a primary endpoint of cognitive function using a rigorous battery of cognitive tests including the HVLT-R, controlled oral word association (COWA) test, Trial-making test (TMT) A and B, and Grooved Pegboard Test. Cognitive deterioration was defined as a decline of more than one standard deviation from baseline in at least one cognitive test. There was less cognitive deterioration at 3 months after SRS alone compared with SRS plus WBRT (63.5 vs. 91.7%, *p* < 0.001). Importantly, cognitive deterioration was also assessed at 12 months in long-term survivors, and the difference in cognitive decline persisted (60 vs. 94.4%, *p* = 0.04) (24). A subsequent study from the NCCTG, also reported by Brown et al. and using a similar cognitive testing battery, compared WBRT vs. SRS to the surgical cavity in patients with resected brain metastases. This study reported a decrease in cognitive-deterioration-free survival with WBRT (3.7 vs. 3.0 months, HR 0.47, 95% CI 0.35–0.63, *p* < 0.0001), as well as an increase in 6-month cognitive deterioration among patients that received WBRT (52 vs. 85%, *p* < 0.001). Consistent with the aforementioned studies, there was no difference in OS (median 12.2 months for SRS vs. 11.6 months for WBRT, HR 1.07, 95% CI 0.76–1.50, *p* = 0.70) (25).

Together the randomized trials above have detailed consistent improvements in CNS control rates with WBRT that do not translate into OS benefits, but are associated with objective declines in cognitive performance. Notably, an unplanned subgroup analysis of the Japanese trial by Aoyama et al. suggested that WBRT might improve OS in a subgroup of patients of patients with favorable prognoses; however, separate secondary analyses from both the NCCTG and EORTC trials have since refuted this finding (27–29). Moreover, a meta-analysis of three of these trials reported by Saghal et al. found no benefit in OS overall and, provocatively, suggested a decrement in OS with WBRT among patients <50 years of age (30). The apparent disconnect between improved CNS control without an accompanying improvement in OS with WBRT may be attributable to the observation that most contemporary patients with brain metastases do not die of CNS progression (4, 31), and that subsequent CNS progression events are often salvageable without WBRT when identified in the context of brain MRI surveillance (32). In response to the consistency of these data, the contemporary NCCN CNS guidelines advocate SRS alone as the preferred treatment for limited brain metastases (5). These guideline recommendations underscore the clinical importance of cognitive decline after WBRT, and suggests that improved CNS control in the absence of an OS benefit fails to justify routine administration in patients with limited CNS disease (5).

One of the largest analyses of the cognitive impact of PCI was reported by Gondi et al. who performed a pooled analysis of the RTOG 0212 and 0214 trials (33). The RTOG 0212 enrolled patients with LS-SCLC who achieved a response to 1st-line therapy and randomized them to PCI with 25 vs. 36 Gy, while the RTOG 0214 was a trial in stage III non-small cell lung cancer (NSCLC) patients who had completed curative-intent therapy and then were randomized to PCI vs. observation (34, 35). In the pooled analysis comparing PCI vs. no-PCI outcomes, declines in tested cognitive function were observed at both 6 and 12 months, and a more than three-fold decrease in patient-reported cognitive outcomes were reported with PCI (33). Moreover, a dedicated analysis of the RTOG 0212 demonstrated increased cognitive decline with higher PCI radiation doses, and the RTOG 0212 and intergroup trials found greater declines in cognition and QOL after PCI in association with older patient age (35, 36).

**Figure 1:**
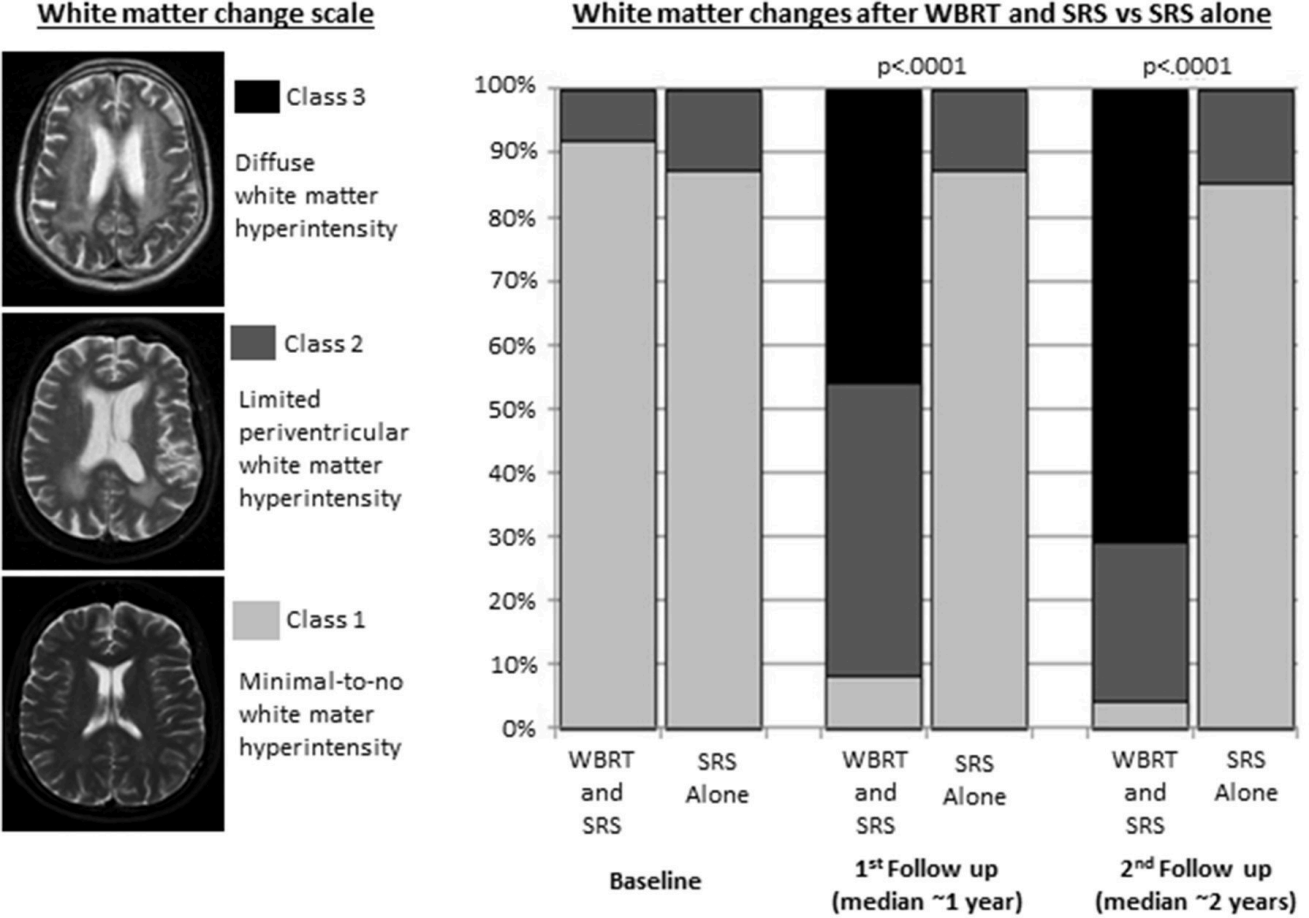
White matter changes in patients with non-small cell lung cancer brain metastases treated with whole brain radiation therapy (WBRT) and stereotactic radiosurgery (SRS) (*n* = 37) or SRS alone (*n* = 31). Adapted from Monaco et al. (39) with permission from the publisher.

The consistent neurocognitive effects of WBRT and PCI are also accompanied by a variety of characteristic anatomic and pathophysiologic correlates. Moderate doses of radiation to the entire brain common to WBRT and PCI have been associated with cortical thinning, demyelination, attenuated capillary density, damage to the vascular endothelium, disruption of the blood-brain barrier, oxidative and pro-inflammatory stress, and impairment of neurogenesis (28, 37–42). In a notable illustrative study, Monaco et al. analyzed longitudinal brain MRI findings in lung cancer patients treated SRS plus WBRT vs. SRS alone and found dramatic increases in the incidence and severity of white matter changes at 1 and 2 years among patients who received WBRT (Figure 1) (39).

## Attenuating the neurocognitive effects of WBRT with pharmacotherapy

For patients requiring WBRT, there has been interest in the use of neuroprotective drugs to preserve cognitive function. Memantine is an antagonist of the N-methyl-D-aspartate (NMDA) receptor, which has important roles in learning and memory. In the setting of vascular dementia, ischemia is associated with excessive NMDA receptor activation and excitotoxicity, and inhibition of the NMDA receptor with memantine represents a neuroprotective strategy (1, 43–45). The RTOG 0614 explored the hypothesis that memantine could be protective in the setting of radiation-induced excitotoxicty and neurocognitive decline. This study was a randomized controlled trial in patients undergoing WBRT for brain metastases, of placebo vs. memantine concurrent with WBRT and for an additional 6 months. Memantine was well tolerated and although the trend toward delayed recall (the primary endpoint) did not reach statistical significance (*p* = 0.059), memantine did delay time to cognitive decline and reduced the rate of decline in memory, executive function, and processing speed (1). As a result, the NCCN CNS and small-cell lung cancer guidelines acknowledge the potential role of memantine to promote cognitive preservation for patients undergoing both WBRT and PCI, although the latter has not yet been tested in a randomized control trial (5, 26).

A separate phase II trial enrolling patients treated with partial brain radiation or WBRT (66% with primary brain tumors, 26% with brain metastases, 8% receiving PCI) randomized 198 patients to placebo or donepezil, a reversible acetylcholine esterase inhibitor. Although donepezil did not improve cognitive composite scores (the primary endpoint), donepezil did result in modest improvements in memory (46). Donepezil, however, is not advocated for cognitive preservation in the context of brain radiation by the contemporary national guidelines (5, 26). In addition to memantine and donepezil, there is lower-level clinical and pre-clinical evidence investigating a variety of other pharmacologic agents (47). For example, one single-arm phase II study evaluated the botanical agent, *Ginkgo biloba*, in 34 patients receiving partial or whole brain radiation and reported improved neurocognitive function assessments over time (48).

Overall, the improved cognitive preservation with pharmacotherapy in the randomized RTOG 0614 represents a unique success in the radiation oncology literature, demonstrating proof of principle that radiation-induced cognitive decline can be attenuated with pharmacotherapy. It is also important to acknowledge, however, that the rates of cognitive decline after WBRT in the RTOG 0614 study remained suboptimal (cognitive preservation at 24 weeks was 31% with memantine vs. 20% with placebo), and further research into novel neuroprotective agents is warranted.

## Reducing WBRT toxicity anatomically: hippocampal-avoidance

A separate strategy to potentially mitigate neurocognitive toxicity in patients undergoing WBRT and PCI involves a reduction in radiation exposure to the hippocampus using conformal intensity-modulated radiation therapy (IMRT). It has been proposed that injury to the neural stem-cell compartment of the hippocampal dentate gyrus may represent an important pathophysiologic mechanism of radiation-induced cognitive decline (2, 49, 50). Providing preliminary data in support of this hypothesis, the multi-institutional single-arm phase II RTOG 0933 demonstrated superior cognitive preservation on the HVLT-R with hippocampal avoidance WBRT (HA-WBRT) as compared to historical WBRT controls (2). As a result, two separate NRG Oncology trials have been launched to evaluate the impact of HA-WBRT in the randomized setting. The phase III NRG CC001 (NCT02360215) is randomizing patients requiring WBRT for brain metastases from various histologies to HA-WBRT vs. conventional WBRT, with a primary endpoint of cognitive preservation on a testing battery including the HVLT-R, COWA, and TMT A and B. All patients in this trial will receive concurrent and adjuvant memantine for 6 months. The phase II/III NRG CC003 (NCT02635009) is randomizing patients with LS and ES-SCLC to PCI with and without hippocampal avoidance, with optional memantine administration. The phase II portion is designed to confirm a non-inferior 12-month intracranial relapse rate with HA-PCI vs. conventional PCI, and the phase III portion will test whether HA-PCI can reduce the rate of 6-month deterioration on the HVLT-R delayed recall.

## The expansion of SRS and narrowing of WBRT indications

While WBRT remains an appropriate treatment for contemporary patients with diffuse brain metastases, there is a wealth of randomized evidence indicating that avoiding WBRT in favor of SRS for suitable candidates can offer superior cognitive preservation and equivalent OS (21–25). SRS alone does, however, come at the cost of higher rates of new brain metastases and greater need for subsequent brain treatments, often in the form of further SRS (4, 21–24, 32). This trade-off between superior cognitive preservation but higher rates of retreatment after SRS has largely been accepted for patients with limited brain metastases, and is now increasingly being studied and supported for patients with ≥4 brain lesions (4, 5, 32).

The strongest current evidence in support of SRS for multiple metastases comes from a Japanese single-arm, multi-institutional prospective study of SRS alone in 1,194 patients with 1–10 brain metastases reported by Yamamoto et al. (4, 51) This study stratified patients into groups of 1, 2–4, and 5–10 brain lesions. OS was superior among patients with a single brain lesion. The key finding, however, was that there were no significant differences in OS, toxicity, or subsequent CNS failure rates among patients with 2–4 vs. 5–10 brain lesions. Moreover, the rates of death from causes related to CNS progression were similarly low (6–10%) in all three cohorts (4). A recent follow up analysis to this study also found no differences in cognitive preservation rates between the cohorts; although, it should be acknowledged that this analysis was limited by its reliance on the mini-mental status exam, which is known to be a less sensitive metric for radiation-induced cognitive deterioration (51). Historically, SRS alone has been considered a reasonable strategy for 1–3 or 4 lesions based primarily on the inclusion criteria of the aforementioned randomized trials of SRS with and without WBRT (21–24); however, this large prospective trial by Yamamoto et al. suggests that SRS for 5–10 brain metastases may be just as safe and effective as SRS for 2–4 lesions, where SRS alone is already widely accepted (4). In response to this data, the NCCN now acknowledges the use of SRS alone as an option for carefully selected patients with “extensive” (a strict number criteria has been intentionally omitted) brain metastases (5).

Along the lines of the Japanese data, our group from the University of Colorado recently reviewed the outcomes of patients with ALK and EGFR driven NSCLC brain metastases treated with SRS alone for ≥4 lesions (range 4–26) (32). The median OS was 3 years (4.2 for ALK and 2.4 for EGFR patients), emphasizing the encouraging prognoses and importance of cognitive preservation strategies in these subsets. OS was comparable regardless of the number of SRS courses and number of brain metastases treated either in a single session or overall. The 5-year freedom from neurologic death and freedom from WBRT rates were 84 and 97%, respectively. Of note, the mean hippocampal and whole-brain doses were exceedingly low even among patient treated to more than 10 lesions in a single session (1.2 G and 0.8 Gy, respectively), as compared to representative plans of conventional WBRT (30.3 and 30.9 Gy) and HA-WBRT (10.6 and 31.9 Gy). These dosimetric findings suggest that SRS alone even for numerous metastases may provide superior hippocampal sparing compared to HA-WBRT and that treating multiple lesions with SRS does not equate to *de facto* WBRT from a dosimetric standpoint (32).

These data, along with a variety of other institutional reports (52–55), provide increasing support for SRS in carefully selected patients with multiple brain lesions. Several randomized trials are ongoing or in development to evaluate WBRT vs. SRS in patients with multiple brain metastases (NCT02353000; NCT03550391; NCT01592968; NCT02953717).

While the role of SRS has been expanded for increasing numbers of brain metastases, the accepted indications for WBRT have also begun to shrink for patients with more limited prognoses. The QUARTZ trial enrolled a population of 538 poor-prognosis NSCLC patients (median OS 9 weeks overall) with brain metastases who were not considered candidates for SRS and randomized them to WBRT or best supportive care. This trial found no significant difference in quality-adjusted life years (primary endpoint) or OS, suggesting that omission of WBRT may be a reasonable recommendation in this population (56).

## Treating brain metastases with CNS-active systemic therapies

Historically, for patients with metastatic disease, the CNS and extra-CNS have largely been viewed as distinct compartments, at least in terms of anticipated response rates to systemic therapy. This division has primarily been attributed to the blood brain barrier, which can reduce conventional chemotherapy concentrations in the CSF to levels much lower than the peripheral blood, making the CNS a potential pharmacologic sanctuary for disease progression. As result, strategies for spatially cooperative combined-modality therapy emerged, with systemic therapy being used conceptually for extra-CNS control and radiation for the treatment of the brain. These historic lines of distinction, however, are now beginning to blur as emerging molecularly-targeted and immunotherapy agents have begun demonstrating encouraging CNS response and control rates in prospective trials (8–20). Below we highlight some of the recent data with an emphasis on some contemporary studies in lung cancer and melanoma (Table 1).

**Table 1:** Selected studies supporting CNS efficacy for systemic agents in melanoma, lung, and breast cancer.

**References**	**Eligibility**	**No. of pts**	**Drug**	**Methods**	**Outcomes**
Margolin et al. 9	Metastatic melanoma with BM (divided into cohorts for symptomatic or asymptomatic)	72	Ipi	Phase II	CNS disease control: 24% in asymptomatic cohort 10% in symptomatic cohort
Goldberg et al. 13	Untreated asymptomatic BM from melanoma or NSCLC	36	Pembro	Phase II	CNS response: melanoma: 22% NSCLC: 33%
Long et al. 19	Untreated asymptomatic melanoma BM with no previous local brain therapy	79	Nivo OR Ipi/Nivo	Randomized Phase II	CNS response: Ipi/Nivo: 46% Nivo: 20% Nivo (after failed local therapy, symptomatic, or with LMD): 6%
Davies et al. 14	Metastatic melanoma with BM cohorts: (A)BRAF^V600E^/asymptomatic/no prior local brain therapy/ECOG 0/1 (B) BRAF^V600E^/asymptomatic/prior local brain therapy/ECOG 0/1 (C) BRAF^V600D/K/R^/asymptomatic/with or without prior local brain therapy/ECOG 0/1 (D) BRAF^V600D/E/K/R^/symptomatic/with or without prior local brain therapy/ECOG 0/1/2	125	D/T	Phase II	CNS response: (A) 58% (B) 56% (C) 44% (D) 59% Duration of response (median): (A) 6.5 months (B) 7.3 months (C) 8.3 months (D) 4.5 months
Gadgeel et al. 12	ALK-positive NSCLC after prior crizotinib (Pts with measurable CNS disease were pooled from two single-arm phase II studies)	50 pts with measurable CNS lesions	Alectinib	Pooled analysis of 2 Phase II studies	CNS response: 64.0% Duration of response (median): 10.8 mo
Peters et al. 17	Previously untreated advanced ALK-positive NSCLC	Total: 303 BM: 43 pts with measurable CNS lesions	Crizotinib OR alectinib	Phase III	CNS response: crizotinib: 50% alectinib: 81% Duration of response (median): crizotinib: 5.5 months alectinib: 17.3 months
Goss et al. 15	T790M-positive advanced NSCLC after progression on other EGFR-TKI with >1 measurable CNS lesion (pooled analysis of two phase II trials)	50	Osi	Pooled analysis of 2 Phase II studies	CNS response: 54.0% Duration of response (median): NR Est duration of response: 75% at 9 mo
Wu et al. 20	T790M-positive advanced NSCLC after progression on other EGFR-TKI. Planned subgroup analysis of AURA3 for patients with baseline CNS lesions.	46 pts with measurable CNS lesions	Osi	Planned subgroup analysis of phase III	CNS response: osimertinib: 70% Platinum-pemetrexed: 31%
Camidge et al. 18	ALK-positive NSCLC (Exploratory analysis of pts with baseline brain metastases from two prospective studies): (1) phase I/II (NCT01449461) (2) phase II ATLA (NCT02094573) arm A (3) phase II ATLA (NCT02094573) arm B	Measurable (>10 mm) (1) 15 (2) 26 (3) 18	brigatinib	Exploratory analysis of a phase I/II and subsequent phase II study	CNS response (among pts with measurable (>10 mm) brain metastases: (1) 53% (2) 46% (3) 67%
Lin et al. 8	HER2+ breast cancer after prior trastuzumab and progressive BM after prior WBRT or SRS	242	L	Phase II	CNS response: 6% (20% in patients on capecitabine-lapatinib expansion)
Bachelot et al. 10	HER2+ breast cancer with BM not previously treated with WBRT, capecitabine, or lapatinib	45	X/L	Phase II	CNS response: 65.9%
Krop et al. 11	Her2+ breast cancer after prior trastuzumab and a taxane (exploratory analysis of EMILIA limited to patients with pre-existing BM)	95	TDM-1 OR X/L	Exploratory analysis of Phase III study	CNS progression: TDM-1: 22.2%; XL: 16.0% Median overall survival: TDM-1: 26.8 mo; XL: 12.9 mo

*BM, brain metastases; L, lapatinib; X, capecitabine; X/L, capecitabine and lapatinib; TDM-1, trastuzumab emtansine; Ipi, ipilimumab; Pembro, pembrolizumab; Nivo, nivolumab; LMD, leptomeningeal disease; D, abrafenib; T, trametinib; Osi, osimertinib; pem, pemetrexed; platinum, cisplatin or carboplatin; NR, not reached*.

In ALK gene-rearranged lung cancer, a pooled analysis of two single arm phase 2 studies of alectinib, with a median follow-up of 12.4 months, demonstrated objective CNS response rates of 64% in patients with measurable CNS disease, and a median duration of response of 10.8 months (12). In a phase 3 study that randomized patients with ALK-rearranged NSCLC to alectinib vs. crizotinib, the CNS response rate and median duration of response for patients with baseline CNS metastases was 81% and 17.3 months, vs. 50% and 5.5 months, for alectinib vs. crizotinib, respectively (17). Similarly, in an exploratory analysis of 2 trials of brigatinib for ALK-positive NSCLC, the objective response rates were 53, 46, and 67%, in patients with measurable brain metastases, from the phase I/II study, ALTA arm A (brigatinib 90 mg daily), and ALTA arm B (brigatinib 180 mg daily), respectively (18). In patients with EGFR TKI-sensitive lung cancer, a pooled analysis of two phase II trials of osimertinib demonstrated CNS response rates of 54% in patients with measurable CNS disease; the median duration of response was not reached with 75% of patients estimated to remain in response at 9 months (15). Similarly, 46 patients included in the AURA3 randomized study of osimertinib or platinum-pemetrexed, had baseline measurable brain metastases. The CNS response rate was 70% in patients randomized to osimertinib vs. 31% in those randomized to platinum-pemetrexed (20).

In BRAF-mutated melanoma, a phase II study of dabrafenib and trametinib for patients with brain metastases demonstrated intracranial response rates of 44–59% in cohorts stratified by BRAF mutation type, prior CNS therapy, symptoms, and performance status. Importantly, however, the durability of response appeared to be suboptimal, with median durations of CNS response of only 4.5–8.3 months across the cohorts (14).

In evaluation of single-agent vs. combination immunotherapy, a randomized phase 2 study of patients with melanoma brain metastases reported objective intracranial responses with a median of 17 months follow-up in 16 of 35 (46%) patients treated with ipilumumab/nivolumab and 5 of 25 (20%) treated with nivolumab alone (19). A separate single-arm, single-institution phase II study of pembrolizumab enrolled patients with untreated brain metastases and reported response in 4 of 18 (22%) patients with melanoma in 6 of 18 (33%) with NSCLC, which appear similar to expected extracranial response rates (13). Intracranial responses were also generally durable, with all but one patient showing continued response at a median of 11.6 and 6.8 months of follow-up in the melanoma and NSCLC cohorts, respectively (13).

The emerging data on systemic agents with enhanced CNS activity are encouraging and have generated appropriate optimism regarding the expanding arsenal for the treatment and prevention of brain metastases. It is important to acknowledge, however, that there is limited prospective data comparing CNS-penetrant agents to strategies incorporating CNS radiotherapy. One recent trial compared icotinib alone, a first generation EGFR-TKI with modest CNS activity, to radiation and chemotherapy for brain metastases and found improved CNS control outcomes with icotinib (57). This trial was notably limited by a lack of detailed information of CNS failure patterns (e.g., existing vs. new lesions) and the use of a non-standard control arm of 1st line chemotherapy in EGFR-sensitive NSCLC. It is probable in this setting, and many others, that a strategy incorporating CNS active agents with a combination of radiation therapy would offer superior CNS disease control outcomes to either therapy alone, and some cautionary retrospective analyses have been reported to that end (58). Moreover, while it may be presumed that drugs with increased activity across the blood-brain barrier will have a lesser impact on cognition than therapies like WBRT, high-level evidence is still lacking. In addition, drugs with prospective data characterizing encouraging objective CNS response rates are still only applicable for a subset patients with metastatic cancer. Nevertheless, the CNS activity of emerging systemic agents is already relevant to contemporary practice and should open the door to new strategies to improve both CNS control and cognitive preservation. Future trials will be needed to assess optimal multidisciplinary integration of local and systemic therapy for brain metastases.

## Evolving CNS management strategies in small-cell lung cancer (SCLC)

Although SRS alone for limited brain metastases has been accepted across most histologies, SCLC represents a notable exception where WBRT remains a guideline recommendation in cases ranging from diffuse to solitary CNS lesions, as well as in patients without radiographic brain metastases in the form of PCI (26). Historic objections to the use of SRS in SCLC have generally included the concern for diffuse interval CNS progression and the potential for a resulting decrease in survival in such cases. There is, however, growing evidence to suggest that SRS alone may be appropriate for some patients with SCLC (Table 2) (32, 59–62). Notably, Serizawa et al. compared the outcomes of SCLC (*N* = 34) and NSCLC (*N* = 211) patients with brain metastases treated with SRS alone and found comparable rates of OS, CNS control, and neurologic mortality in SCLC and NSCLC patients (59). Yomo and Hayashi reported on 70 SCLC patients treated with SRS (46 patients underwent SRS alone without prior PCI or WBRT), with median OS of 7.8 months and encouraging one- and two-year neurologic mortality-free survival of 94 and 84%, respectively (61). Recently, our group reported a National Cancer Database (NCDB) analysis of upfront SRS (*N* = 200) vs. WBRT for brain metastases, and observed favorable survival outcomes with SRS overall and on propensity-score matched analyses (6). While these retrospective data are subject to confounding from selection bias, they do suggest that a subset of patients with SCLC might be safely and effectively managed with SRS alone and point to the need for prospective investigation. One recently opened randomized phase II trial (ENCEPHALON) is comparing SRS to WBRT for SCLC patients with 1–10 brain metastases (NCT03297788).

**Table 2:** Studies of first-line SRS (no prior PCI or WBRT) for SCLC brain metastases.

**References**	**No. of patients**	**Methods**	**Outcomes**
Serizawa et al. 59	34 (compared with 211 NSCLC pts)	Retrospective comparison of SRS outcomes for SCLC vs. NSCLC	No significant difference in any outcome, including local control, overall survival, and neurologic survival
Jo et al. 60	50 (first-line SRS: 12)	Retrospective	Median overall survival for first line SRS group: 4.6 months
Yomo and Hayashi 61	70 (first-line SRS: 46)	Retrospective	Median overall survival: 7.8 months One-year neurologic death-free survival: 94% Two-year neurologic death-free survival: 84%
Ozawa et al. 62	94 (LS-SCLC, managed with strategy of PCI omission, MRI surveillance, and SRS salvage)	Retrospective	Median overall survival: 34 months 30.8% of patients developed brain metastases within 2 years of diagnosis ^*^No significant difference in outcomes when compared to 29 patients that received PCI
Robin et al. 6	200	Retrospective/US national cancer registry database	Median overall survival: 10.8 months ^*^Compared with matched cohort of patients that received WBRT, superior OS observed with SRS

*SCLC, small-cell lung cancer; NSCLC, non-small-cell lung cancer; PCI, prophylactic cranial irradiation*.

For patients with a response to first-line therapy, PCI remains a guideline endorsed therapy for LS-SCLC patients and a treatment option for those with ES-SCLC (26). PCI was accepted in SCLC management after a 1999 meta-analysis of 7 trials of primarily LS-SCLC patients (86%) reported a 5% improvement in OS at 3 years, and a subsequent 2007 EORTC randomized trial in ES-SCLC reported a 14% OS benefit at 12-months and a 1.3 month (6.7 vs. 5.4) improvement in median survival (63, 64). The OS advantage of PCI in the contemporary MRI era, however, was recently challenged by a phase III randomized trial in Japan that, unlike the EORTC or trials included in the aforementioned meta-analysis, required brain MRI staging and surveillance (every 3 months in year-1 and every 6 months in year-2) (7). This trial found a similar reduction in brain metastases to prior PCI studies, but reported no difference in PFS and, provocatively, a trend toward improved OS (median 13.7 vs. 11.6 months) with omission of PCI (7).

Reconciling the conflicting OS outcomes from the ES-SCLC PCI trials from the EORTC and Japan requires some consideration of the key differences in their respective designs. First, the Japanese trial mandated MRI staging, whereas the EORTC trial only obtained CNS imaging for neurologic symptoms. It is estimated that up to 25% of SCLC may have brain metastases when staged with MRI at diagnosis (65), and one study found that up to one-third without brain metastases developed them during first-line therapy (66). Thus, a meaningful but unknown percentage of patients in the EORTC trial were actually randomized to WBRT for brain metastases vs. observation until symptoms. Second, the MRI surveillance in the Japanese trial allowed for more patients to receive salvage radiation, presumably because metastases were identified at earlier and, thus, more treatable stages. Among patients who ultimately developed brain metastases in the no-PCI arms of these trials, 83% successfully underwent salvage radiation in the Japanese trial vs. only 59% in the EORTC study (7, 64). Additionally, it is important to note that, in all, only 58% of patients in the no-PCI arm of the Japanese trial (64 of 111 total patients) ultimately required brain radiation and a clinically meaningful 42% did not (7), indicating that the trial was not simply comparing early vs. late radiation, as has sometimes been a suggested.

Overall, the results of the Japanese ES-SCLC trial are important to contemporary clinical practice because they suggest that (1) brain metastases identified earlier in the context of MRI surveillance may be salvaged without negatively impacting survival and (2) that a meaningful subset of patients with SCLC who do not develop brain metastases can be spared the neurocognitive sequela of PCI altogether. Moreover, the design of the Japanese trial also points to the need for new studies in LS-SCLC comparing PCI to MRI surveillance strategies, as the trials included in the 1999 meta-analysis of LS-SCLC were all in the pre-MRI era and the majority of patients did not undergo brain CT staging or surveillance in those studies (67, 68). In response to this data, the NCCN has now changed PCI from recommended to optional in ES-SCLC and has endorsed MRI surveillance for any patient that does not receive PCI (26).

## Conclusions and future directions

The management of brain metastases remains a complex and highly-individualized discipline in oncology. As prognoses continue to improve for patients with brain metastases, efforts to minimize the cognitive sequelae of therapy will only become increasingly important. Numerous clinical trials have characterized the deleterious effects of moderate doses of radiation to the entire brain common to WBRT and PCI, challenging investigators to develop new strategies to attenuate, avoid, or delay the neurocognitive effects of these therapies. Pharmacotherapy and anatomic avoidance strategies are actively being investigated, as is the expansion of SRS candidacy to patients with increasing burdens of CNS disease. It is also clear that management of brain metastases will become increasingly multidisciplinary in the context of emerging systemic agents with enhanced CNS activity. A new generation of combined-modality trials involving local and systemic therapies will be needed to evaluate the optimal strategies for durable CNS disease control, neurocognitive function, and survival in the rapidly evolving landscape of therapies for metastatic disease.

## Author contributions

TR and CR: Conception and design, writing, and final approval of manuscript.

### Conflict of interest statement

The authors declare that the research was conducted in the absence of any commercial or financial relationships that could be construed as a potential conflict of interest.
